# Multimorbidity, disadvantage, and patient engagement within a specialist homeless health service in the UK: an in-depth study of general practice data

**DOI:** 10.3399/bjgpopen17X100941

**Published:** 2017-10-04

**Authors:** Anton B Queen, Richard Lowrie, Janice Richardson, Andrea E Williamson

**Affiliations:** 1 Medical Student, University of Glasgow, Glasgow, Scotland, UK; 2 Lead Pharmacist Research and Development, Pharmacy and Prescribing Support Unit, NHS Greater Glasgow and Clyde, Glasgow, Scotland, UK; 3 Research Manager, Pharmacy and Prescribing Support Unit, NHS Greater Glasgow and Clyde, Glasgow, Scotland, UK; 4 Clinical Senior University Teacher, General Practice and Primary Care, School of Medicine, University of Glasgow, Scotland, UK

**Keywords:** homeless persons, chronic disease, delivery of healthcare, vulnerable populations

## Abstract

**Background:**

There is a paucity of current health data regarding users of a specialist homeless health service in the UK.

**Aim:**

To describe the health of users of a specialist homeless health service by assessing levels of multimorbidity, social exclusion — by measuring severe and multiple disadvantage (SMD) — and patient engagement with health care.

**Design & setting:**

Analysis of patient-level data from computerised records of patients registered with a specialist homeless health service in Glasgow, Scotland.

**Method:**

Data for 133 patients were extracted using a data extraction form. Multimorbidity and SMD were described using categorisation adapted from previous literature in this field. Stepwise regression analysis was carried out to assess the relationship between domains of SMD experienced and the number of long-term conditions (LTCs) a patient had.

**Results:**

The average age of patients in the cohort was 42.8 years, however levels of multimorbidity were comparable to those aged ≥85 years in the general population. The average number of LTCs was 2.8 per patient, with 60.9% of patients having both mental and physical comorbidity. SMD was categorised into three domains: homelessness; substance misuse; and previous imprisonment. More than 90.0% of patients experienced ≥2 domains of SMD, and SMD experiences were associated with multimorbidity: as domains of SMD experiences increased, so did the number of LTCs a patient was recorded as having.

**Conclusion:**

This cohort of patients has a complex burden of health and social care needs, which may act as barriers in the provision of effective health care.

## How this fits in

This in-depth study of general practice data, for users of a specialist homeless health service in the UK, is the first to describe multimorbidity in a UK homeless population. It also assesses the relationship between deep social exclusion and multimorbidity, and confirms that deep social exclusion is not only common in a homeless population, but also associated with experiencing a greater number of LTCs. This approach quantifies experiences of social exclusion and helps practitioners to understand why providing effective care for users of homeless health services is complex and challenging. These insights might enable services to be tailored so they better meet patients’ needs.

## Introduction

Homelessness remains an issue across the UK.^1,2^ Homelessness has a detrimental impact on an individual: homeless people have poorer health than the general population and experience premature mortality.^3–5^ Furthermore, homeless people are likely to experience multimorbidity (the presence of ≥2 LTCs),^6–8 ^which is associated with a poorer quality of life and described as a challenge to the healthcare system.^8^ In spite of this, published data regarding multimorbidity in a homeless cohort are limited.

People who are homeless are high service users of primary and secondary care,^4^ and have complex health and social care needs.^9^ Literature from the social sciences has described many homeless people as experiencing multiple exclusion homelessness (MEH) and SMD — measures of deep social exclusion.^9,10^ MEH describes an individual who is homeless and has experienced another form of deep social exclusion, including time spent in institutional care, misusing substances, or taking part in 'street culture activities'; such as 'begging, drinking on the streets, and sex work'. Poor mental health and substance misuse often precede homelessness in this population.^9^

SMD can be used to assess deep social exclusion and MEH in a population. People are described as having SMD if they experience any one of the following:

- imprisonment;
- substance-misuse issues; and
- homelessness.

These experiences overlap and interact, pushing an individual into the most extreme margins of society.^10^ Research has shown there is a high degree of intersectionality between these experiences, in that they rarely occur in isolation in any one individual and they require input from multiple health and social care services. Experiencing SMD is detrimental to health: individuals are likely to report a poorer quality of life than other socially disadvantaged groups, face greater stigma, and prove challenging to provide with effective healthcare interventions.^10^

There is felt to be a large evidence gap regarding individuals who fall into the category of SMD.^10^ Evidence suggests that experiencing more than one form of deep social exclusion is common.^10^ Despite knowledge of the prevalence and adverse impact of SMD and MEH, it is yet to be described in the health literature.

The Glasgow Homeless Health Service (GHHS) was set up in 2003 to meet the needs of the homeless population within the NHS Greater Glasgow and Clyde catchment area, which includes 1.2 million inhabitants. GHHS offers a variety of health and social care services onsite, including a dedicated GP service. The homeless practice is used by more than 700 permanent and temporary registered patients per year, with the majority being temporarily registered. The level of care given is the same, regardless of registration status.

Most patients use the specialist GP service a handful of times then move to a mainstream practice, so permanent registration is unnecessary. The homeless practice offers a mixture of pre-booked and drop-in appointments lasting 15 minutes, and an outreach service to accommodation projects across the city. One of the aims of the homeless practice is to encourage patients to move back to mainstream services once they have moved on from crisis homelessness.

The most recent study conducted in a specialist homeless health service in the UK uses data from 2009.^5^ With a growing body of social science and health literature published since this time, this study aimed to ascertain what can be learned for future research and service design using contemporary measures of multimorbidity and extreme social exclusion in addition to traditional data about patient demographics, health diagnoses, and service utilisation.

## Method

Patient-level data from general practice records of all permanently registered patients at GHHS as of 15 October 2015 (*n* = 133) were analysed. This study was restricted to permanently registered patients at GHHS as Caldicott Guardian permission for temporary registered patients’ data is held by the GP practice where they are permanently registered.

Patients who are permanently registered at GHHS tend to:

- have health needs that require secondary care input — permanent registration allows informational continuity; 
- be deemed especially vulnerable for a variety of reasons, including mental health needs;
- be likely to have been homeless for a prolonged period of time; and
- ask to become permanently registered. 

Information was gathered from two GP data sets: EMIS and Docman. EMIS is an electronic database used to store clinical information; Docman is an electronic record of correspondence with secondary and social care. If patients were deceased or had de-registered from practice, during the study period, certain data became inaccessible and was not collected. However, deceased and de-registered patients were included in analyses. 

Demographics, recent clinical markers, prescribing records, and acute health conditions were recorded using a data extraction template. LTCs and multimorbidity were recorded using the categorisation described by Barnett *et al*.^8^ Multimorbidity was defined as the presence of ≥2 LTCs, while SMD was measured based on methods adapted from Bramley *et al*.^10^ Box 1 gives examples of data variables and their criteria.

**Box 1.** Collected data

Variable	Data collected	Data source
Demographics	Age	Date of birth stored in EMIS
	Ethnicity	Patient registration document stored in Docman
	Accommodation type	Accomodation type recorded on patient registration document
	Registration with mainstream GP in 2015	Stored in EMIS
	Recorded period in prison in 2015	Correspondence with prison service in 2015 in EMIS/Docman
Clinical marker	Smoking status	Stored in EMIS or patient registration document
	Body mass index	Calculated from height and weight in EMIS record, if available
	Blood pressure	Stored in EMIS in 2015
Acute health conditions	Alcohol misuse in 2015	Read Codes: 'alcohol misuse', 'alcohol dependence syndrome', 'alcohol problem drinking' in EMIS in 2015
	Drug misuse in 2015	Read Codes: 'Drug dependence', 'injecting drug user', opioid type drug dependence', benzodiazepine dependence', 'hypnotic or anxiolytic dependence', 'cocaine type drug dependence', 'novel psychoactive substance misuse', and 'substance misuse' recorded in EMIS in in 2015
	Skin condition in 2015	Read Code describing any skin condition recorded in EMIS in 2015
Long-term conditions	Barnett *et al* (2012) outlined 40 conditions defined as 'long-term conditions' (LTCs); these were recorded according to their existing criteria and taken into consideration when counting the LTCs. Examples include:	Hypertension: Read Code ever recorded in EMIS
		Depression: Read Code recorded in EMIS, within previous 12 months or ≥4 antidepressant prescriptions (excluding low-dose tricyclics) in 2015
		Painful condition: ≥4 prescription-only analgesic prescriptions in last 12 months or ≥4 antiepileptics in the absence of an epilepsy Read Code recorded in EMIS within previous 12 months
	Certain conditions were not included in the long-term condition count but featured in the data collection. Conditions were chosen as they were considered especially relevant for this population.^3,5,7^ These conditions were:	HIV: Read Code ever recorded in EMIS
		Myocardial infarction: Read Code ever recorded in EMIS
		Back pain: Read Code ever recorded in EMIS
		Hepatitis C virus: positive polymerase chain reaction test for hepatitis C recorded in EMIS. Viral hepatitis was included in LTC count but hepatitis C prevalence was also looked at in isolation
Severe and multiple disadvantage	Bramley *et al* (2015) described three SMD domains imprisonment, homelessness, and problem drug use. This article described patients as SMD1-3 based on the total number of domains of SMD they had experienced. A cumulative total was given for each patient; therefore, an individual experiencing homelessness plus previous imprisonment plus problem drug use would be described as SMD 3. A single experience in one of these domains would qualify an individual as having experienced this domain of SMD	Previous imprisonment: correspondence with prison services recorded in EMIS/Docman
		Homelessness: Read Code ever recorded in EMIS
		Problem drug use: Read Code ever recorded in EMIS
Referrals and engagement	Engagement defined as patient attending referred service. (Record of attendance = engaged; record of non-attendance = did not engage).	Referred to further care: referrals section in EMIS/Docman
		Engagement: Recorded in EMIS and free text in GP consultation notes
Prescribing	Medication prescribed	Acute and repeat prescriptions from 2015 recorded in EMIS
Primary care services	Number of GP encounters within (Glasgow Homeless Health Service) GHHS	Recorded in EMIS in 2015
	Number of nurse encounters within GHHS	Recorded in EMIS in 2015
	Number of pharmacist encounters within GHHS	Recorded in EMIS in 2015
	Number of GP visits to patient accommodation	Recorded in EMIS in 2015
Secondary care services	Number of presentations to accident and emergency (A&E)	Recorded in Docman in 2015
	Number of in-patient hospital admissions	Recorded in Docman in 2015

Collected data were compared with those of with relevant published studies on primary care and homelessness. These included clinically recorded data and patient-reported data (Box 2).

**Box 2.** Characteristics of comparable studies.^3,5,7,8^

Study	Population	Data type
Barnett *et al,* 2012	1.75 million patients registered with a mainstream GP in Scotland	Clinical records
Hewett *et al,* 2011	1000 patients accessing Leicester Homeless Health Service	Clinical records
Homeless Link, 2014	2590 people using homeless services across 19 areas in England	Self-reported
Keogh *et al,* 2015	105 patients using services in Dublin city centre	Self-reported

Descriptive statistical analysis of the data was conducted. Stepwise regression analysis was carried out to assess the relationship between the number of SMD domains experienced (independent variable) and the number of LTCs (dependent variable). Stepwise regression was used to provide a simplified model of the relationship between these two variables, with the least number of predictors.

In this study, drug use was termed both an LTC and a domain of SMD. This was deemed appropriate as, in the context of SMD, drug use is used as a measure of social exclusion as opposed to a health problem. As such, although the measure is the same, the consequences are different. Confidence intervals (CIs) were reported where appropriate and *P*-values of <0.05 were considered to be statistically significant. All analyses were carried out using Minitab (version 17).

## Results

### Demographics

At beginning of study, 133 patients were registered with GHHS. Twenty-three patients (17.3%) were deceased or had de-registered from the practice during the study period. Men accounted for 86.0% of the 133 registered patients within the GHHS, and were aged, on average, 42.8 years. The majority of patients were white Scottish (83.5%) and hostel accommodation was the largest proportion of accommodation type (40.6%) used; nine patients were recorded as sleeping rough in the previous year. A large number of patients (111/133, 83.5%) were current tobacco smokers.

### Long-term health conditions

Long-term physical and mental health problems were common: 63.2% of patients (95% CI = 55.0 to 71.4) had a long-term physical condition and 61.7% of patients (95% CI = 53.4 to 69.9) had a long-term mental health condition.

The average number of LTCs found within the practice was 2.8 per patient (95% CI = 2.53 to 3.22). Only five patients (3.8%) were recorded as having no LTCs, with over three-quarters of people experiencing ≥2 LTCs. There were a significant number of patients who had a diagnosis of both long-term physical and mental health problems (60.9%).

**Figure 1.**
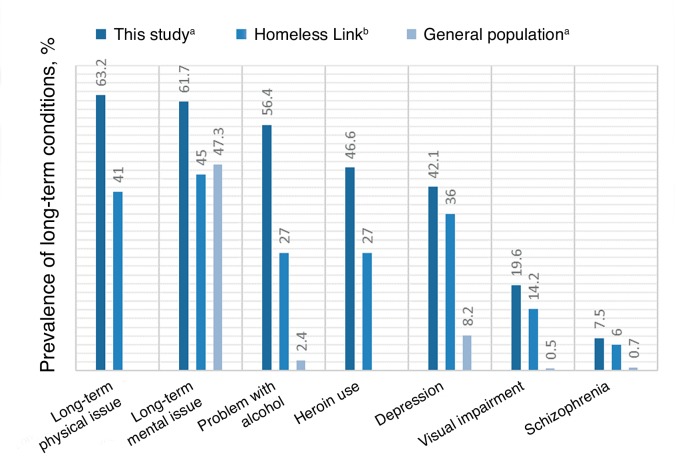
Prevalence of LTCs of high prevalence in homeless cohorts.^3,5,7^ ^a^Clinically recorded data. ^b^Self-reported data. Figures for population-representative sample for long-term mental health problem are based on data for the socioeconomic class of greatest deprivation.

Almost two-thirds (62.4%) of patients were recorded as being addicted to, or having used, illicit drugs. Alcohol was the most common drug of misuse, with 56.4% of patients (95% CI = 48.0 to 64.8) recorded as having issues relating to alcohol. Heroin was the next most commonly used illicit drug (46.6% of patients; 95% CI = 38.1 to 55.1), followed by benzodiazepines (23.3%). Figure 1 outlines the prevalence of selected LTCs.

**Figure 2.**
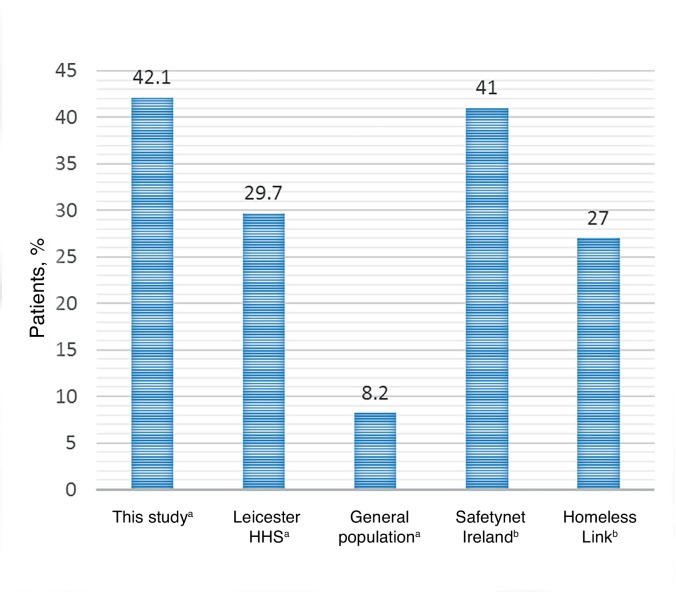
Prevalence of depression in this study versus other homeless cohorts^3,5,7^ and a population-representative sample from general practice records.^8^ ^a^Clinically recorded data. ^b^Self-reported data. HHS = Homeless Healthcare Service.

Mental health diagnoses characterised this particular cohort; two-fifths of patients (42.1%; 95% CI = 33.7 to 50.5) were recorded as having depression. Figure 2 details the prevalence of depression in this study and other homeless cohorts.

One-quarter of patients (26.4%) had a recorded attempt of suicide or self-harm in their medical record. Schizophrenia, non-organic psychosis, and bipolar disorder were prevalent in 7.5% of patients (95% CI = 3.0 to 12.0), while anxiety and other neurotic, stress-related, and somatoform disorders affected a further 7.5% of the cohort.

**Figure 3.**
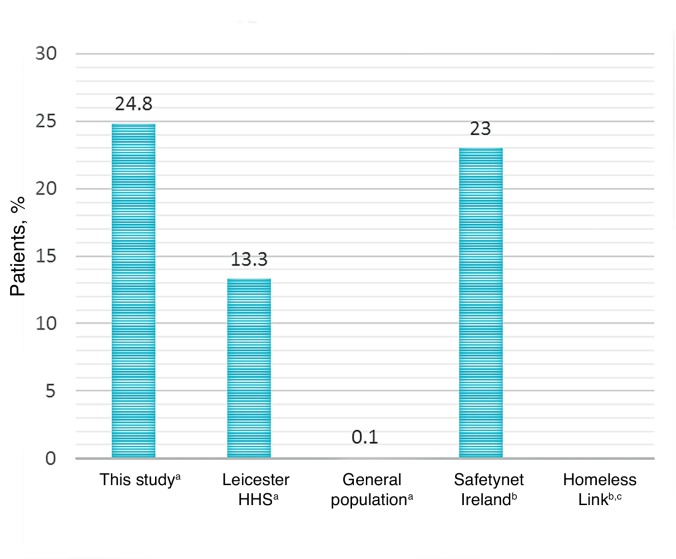
Prevalence of viral hepatitis C in this study versus other homeless cohorts^3,5,7^ and a population-representative sample from general practice records.^8^ ^a^Clinically record data. ^b^Self-reported data. ^c^No data available.

Almost one-quarter of patients (24.8%; 95% CI = 17.5 to 32.1) had a polymerase chain reaction (PCR)-positive hepatitis C infection (Figure 3). Accidents and physical trauma were common, with one-third of patients having experienced a previous head injury (29.3%) or fracture (36.8%).

### SMD

All patients in this study experienced at least one SMD domain. In total, 9.8% experienced homelessness only, 36.8% experienced homelessness and previous imprisonment or substance misuse issues, and 53.4% faced homelessness, previous imprisonment, and substance misuse issues.

**Figure 4.**
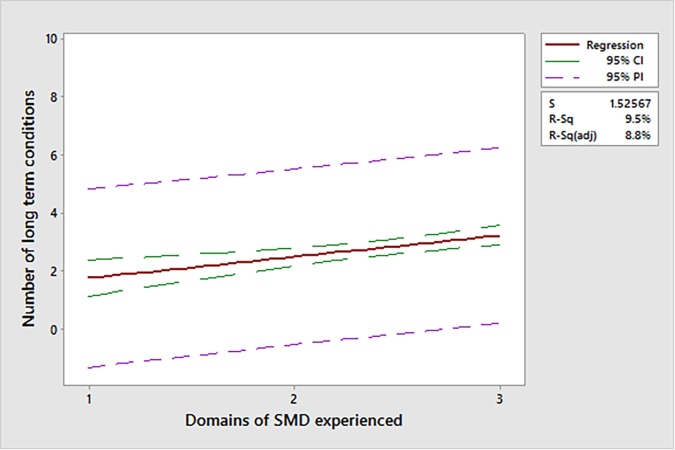
Relationship between domains of severe and multiple disadvantage experienced and long-term conditions. SMD = severe and multiple disadvantage. PI = prediction interval.

Experiences of deep social exclusion were associated with multimorbidity; as SMD experiences increased, the number of LTCs a patient was recorded as having increased (*P*<0.001); Figure 4 depicts this relationship.

### Service utilisation and prescribing

In total, 124 patients (93.2%) had a recorded encounter with a GP in the previous year, with a median of six GP encounters per patient (range 0–32 encounters). More than one-fifth (26.3%) of patients had a recorded encounter with a nurse in the previous year and 25.6% of patients had a recorded encounter with a pharmacist. The median number of encounters with all services in the previous year was seven per patient (range 0–47 encounters).

Almost half of patients (48.1%) attended accident and emergency (A&E) in 2015; of these, more than 95% had a mental health condition, while 70.3% experienced both a long-term mental and physical health condition.

That same year, one-quarter (25.0%) of patients who had had a myocardial infarction were prescribed guideline secondary prevention, two-thirds of patients diagnosed with hypertension had been prescribed antihypertensives, and three-quarters of patients with chronic obstructive pulmonary disorder were receiving treatment.

### Engagement

Most patients who were using illicit or recreational drugs were referred to an addiction service (86.7%); of these, more than half (56.6%) had been recorded as attending that service. Of all patients with alcohol issues, over four-fifths (84.0%) were supported by an addiction service, with nearly half (49.2%) attending that service. Half (50.0%) of all patients with a recorded mental health condition were supported by mental health services, with an uptake rate of 51.2%.

## Discussion

### Summary

Widespread ill health is common in patients who are homeless; users of the specialist homeless health service were likely to experience acute illness and long-term disease. The average age of this cohort was 42.8 years, but the levels of multimorbidity recorded were comparable to those of individuals aged ≥85 years in the general population.^8^ All patients experienced extreme social exclusion, measured by the number of SMD domains experienced. SMD may act as a predictor of multimorbidity: the greater number of domains of SMD experienced, the more LTCs a patient is likely to experience.

High rates of uptake and attendance were found in services colocated in the homeless health service (for example, the addictions and mental health services), suggesting the specialist service is the healthcare facility most visited by the patients in this study. A&E attendance was common.

### Strengths and limitations

Data were collected from general practice records, which hold the most complete set of available health data for a patient. Using both the GP-coded and clinical notes system (EMIS) and the record of health service communication between primary and secondary care (Docman) improved the reliability of findings and allowed a more-detailed picture of a patient’s medical history to be obtained.

As administrative data rely on information being recorded, the information gathered may be incomplete and underestimate the prevalence of conditions. Patient turnover of 17.3% from the beginning of the study period to the time that data collection was completed (October 2015 until February 2016) meant that some data became inaccessible for de-registered or deceased patients. This is likely to have impacted on completeness of data collected for these patients. Furthermore, the numbers of practice nurse encounters were likely to have been underestimated, as a separate system exists within the practice for recording nurse consultations. This study did not have access to this system. This study did not look at primary reason for encounters with the service.

The results presented here provide information on those who are permanently registered as general practice patients with a specialist homeless service, so may have higher rates of engagement than the general homeless population. In addition, those who had registered with the GP were perhaps more likely to need treatment for ill health than those who had not. This may be reflected in the prevalence of conditions within this study, in which symptomatic conditions and associated prescriptions were more common than those that were non-symptomatic.

### Comparison with existing literature

This cohort of patients shared similar demographics and health problems as described in other homeless groups across the UK: the majority of patients were male, with an average age of 42.8 years, and displayed a high prevalence of addiction and mental health problems.^4^ Rates of depression were higher than those found in other homeless populations^3–5,7^ and in the general population.^8^ In addition, rates of attempted suicide and self-harm in the Glasgow cohort were far greater than those found in a similar homeless service in England.^5^ However, excess rates of attempted suicide and self-harm within the GHHS cohort may not be particularly surprising; literature suggests an excess prevalence of these in the Scottish population.^11^

Just over three-quarters (77.4%) of patients in this study were multimorbid, with physical and mental comorbidity being common. Six in every 10 patients have both a physical and mental health diagnosis, compared with only one in 10 in mainstream primary care in Scotland.^8^ Patients visiting GHHS had greater levels of multimorbidity than a similar Australian population,^6^ but lower levels than a similar Irish cohort.^7^ Reasons for this were not explored, but this study used a larger number of conditions to define multimorbidity compared with the Australian study, and work in Ireland relied on patient self-reported data.

The results presented here show that patients visiting GHHS are likely to face deep social exclusion. This study measured deep social exclusion using SMD, allowing MEH to be assessed within the cohort. More than 90.0% of participants had experienced at least two (out of three) domains of SMD, which reflects the intersectionality of MEH experiences within the GHHS cohort that has previously been described in social policy literature.^9^ Individuals who experience deep social exclusion, rarely experience only one form; experiences of homelessness, substance misuse, and previous imprisonment commonly exist together. Along with the findings presented here, evidence suggests that, as experiences of SMD accumulate, so too do complex and interconnected health and social needs of individuals.^10^ These patients are likely to report poorer overall health and, as a consequence of their complex needs, it is often challenging for healthcare providers to deliver effective interventions. This study has shown objective evidence between the relationship of SMD experiences and their consequences on health: accumulation of SMD experiences is likely to result in the accumulation of LTCs.

### Implications for research and practice

By measuring SMD, evidence is presented of the medical and social complexity that comes with seeking to meet the health needs of people who are homeless. Primary care has previously been described as the ideal environment in which to provide treatment for patients with multiple conditions,^8^ but this assertion may not take into account those patients who are facing extreme social exclusion. The high burden of social exclusion within this study's cohort, and the intersectionality of SMD experiences, serves to highlight the challenges faced by primary care providers. This may reinforce the need for a more integrated approach to the management of these patients, in which health and social care services collaborate to address patients’ needs at the same time. Further work utilising the SMD perspective in the healthcare setting may yield new insights into how it may be possible to address long-term health needs and improve engagement with anticipatory care in the homeless population so that morbidity and mortality outcomes can be improved.
